# Design of a Single-Material Complex Structure Anthropomorphic Robotic Hand

**DOI:** 10.3390/mi12091124

**Published:** 2021-09-18

**Authors:** Li Tian, Jianmin Zheng, Nadia Magnenat Thalmann, Hanhui Li, Qifa Wang, Jialin Tao, Yiyu Cai

**Affiliations:** 1 Institute for Media Innovation (IMI), NANYANG Technological University, Research Techno Plaza, XFrontiers Block Level 03-01, Singapore 637553, Singapore; tianli@ntu.edu.sg (L.T.); asjmzheng@ntu.edu.sg (J.Z.); hanhui.li@ntu.edu.sg (H.L.); wang1429@e.ntu.edu.sg (Q.W.); jtao004@e.ntu.edu.sg (J.T.); 2 MIRALab, University of Geneva, Battelle, Building A, 7, Route de Drize, 1205 Geneva, Switzerland

**Keywords:** soft robot materials and design, biomimetic, biologically-inspired robots, customized 3D model, 3D printing

## Abstract

In the field of robotic hand design, soft body and anthropomorphic design are two trends with a promising future. Designing soft body anthropomorphic robotic hands with human-like grasping ability, but with a simple and reliable structure, is a challenge that still has not been not fully solved. In this paper, we present an anatomically correct robotic hand 3D model that aims to realize the human hand’s functionality using a single type of 3D-printable material. Our robotic hand 3D model is combined with bones, ligaments, tendons, pulley systems, and tissue. We also describe the fabrication method to rapidly produce our robotic hand in 3D printing, wherein all parts are made by elastic 50 A (shore durometer) resin. In the experimental section, we show that our robotic hand has a similar motion range to a human hand with substantial grasping strength and compare it with the latest other designs of anthropomorphic robotic hands. Our new design greatly reduces the fabrication cost and assembly time. Compared with other robotic hand designs, we think our robotic hand may induce a new approach to the design and production of robotic hands as well as other related mechanical structures.

## 1. Introduction

The human hand is dexterous as it has a complex and high-efficiency mechanical structure [1]. It consists of the palm and five fingers, which contain 8 wrist bones, 5 palm bones, and 14 finger bones in total. It can be divided into three different layers: the external layer (surface), bone layer (skeleton), and intermediate layer (tissues). From a kinematic point of view, it has 15 movable joints and 21 DOFs (degree of freedom) altogether for the finger part. All of these DOFs are controlled by muscles, tendons, pulleys, tissues, bones, and ligaments. There are three main approaches to duplicate a human hand with a robotic hand.

The traditional approach aims to build hand-shape movable mechanical structures using solid parts such as laser-cut metal or plastic [2]. They usually have rigid or dislocatable joints made of screws or springs [3]. Various tendons or linkage bars are commonly used for transmitting the force created from actuators. The use of many parts will not only lead to a long fabrication duration, but also require a heavy testing procedure.

Unlike the robotic hands mentioned above, the body of a soft robotic hand is made of soft and deformable material. Nature rubber and silicon rubber are two commonly used soft materials in our daily life. Most soft body robotics choose silicon rubber as it has better plasticity. Since 3D-printable soft material has been invented, in the last 5 years, increasingly more 3D-printed artificial organs have appeared for medical purposes [4,5,6,7], as well as for soft robotics [8]. The Young’s modulus value of human hand tissue is around 100 kPa, which is soft [9]. When the human hand grasps an object, the finger contact surface will increase significantly when the finger force increases from 0.1 N to 2 N [10]. Recently, with the understanding of the benefit of soft design, increasingly more 3D-printable soft robotic hands have emerged [11,12]. A typical soft body robotic hand is usually actuated by gas/liquid pressure [13,14,15], driven cable, or “new actuators” such as shape memory alloy (SMA) or artificial muscles [16,17,18]. In 2019, a soft robotic hand, BLC-26, performed six variations of in-hand manipulation, which are translations and rotations along the x-, y-, and z-axes [19]. A recently proposed four-finger soft body robotic hand displayed the ability to unscrew the cap of a jar [20]. Compared with a rigid body robotic hand, a soft body robotic hand easily deforms during grasping or manipulation, which helps to secure the target object [21,22,23]. However, most of them have a complete soft body structure design, causing them to move like invertebrates, or resulting in a weak grip force [11,16,24,25]. Another problem is that all existing actuators such as SMA, pneumatic actuators, and hydraulic actuators cannot fully duplicate the muscle function while taking up the same volume [26]. Two recently invented artificial muscles, “Thin McKibben muscle” and “bubble artificial muscles”, have similar shape and flexibility as natural muscle. They also require air pressure as an input and the efficiency is not as high as that of real muscles [17,18]. For these reasons, these soft robotic hands do not aim at mimicking human hand grasping gestures.

The third approach is the highly biomimetic design, which aims to be “anatomically correct”. The ACT hand designed in 2013 was the first trial for mimicking the real hand on an anatomical level [27]. After that, three new robotic hands were created following a similar design concept [28,29,30]. These designs all have functional parts to simulate human hand bones, ligaments, and tendons, which are made of different materials such as rubber, silicone, or cotton wire. Same as the traditional approach, a complex structure requires high costs in assembly and maintenance. A recently designed robotic hand can be 3D-printed in one go using two different materials. However, it does not have an actuated system such as tendons and pulleys to move actively [31]. Our previous work on a highly biomimetic design can be found in [32,33].

**Figure 1:**
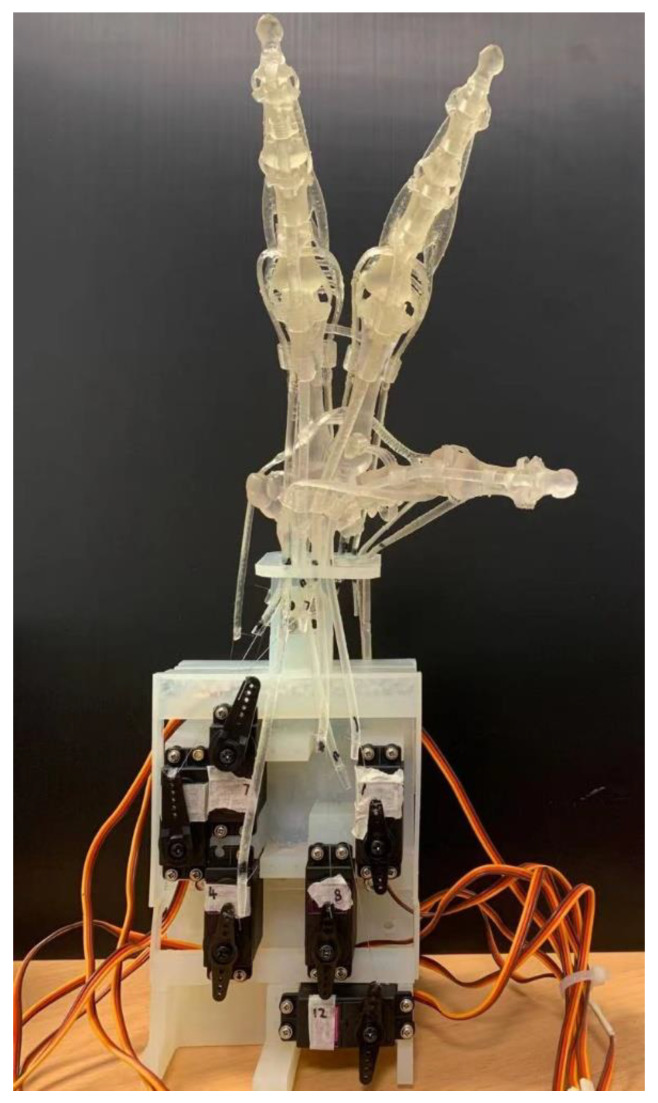
The proposed robotic hand with an actuation system.

The different parts of the human hand have very different physical properties. For example, in hardness, the Young’s modulus of finger bones is 10 GPa, while that of finger tissue is 50 KPa [11]. The hand tendon is not only soft, but also elastic. Therefore, there is no existing solution to modelling and fabricating an articulated robotic hand using a single material. On the other hand, with the rapid growth of 3D printing technology, more and more new 3D printing materials and designs are available for rapid prototypes [4,5,6,7]. Based on the third approach, we proposed a new 3D hand model that aims to 3D print a full DOF robotic hand that can duplicate the most of the functionality of the human hand. It has a biological joint design that contains human-like artificial bones and ligaments. It also has a biomechanical transmission system. To verify our method, we 3D-printed the three-finger robotic hand and tested it with an actuation system with 12 electrical servo motors. As shown in Figure 1, our method resulted in a highly human-like design. It could be used separately for grasping study or installed onto a humanoid robot. As it has a structure and motion range close to that of a real human hand, it also could help further research in the medical or biological fields. The hand part is 3D-printed within 10 h with around 100 mL of 3D printing material, which is worth around US$50. The assembly time is less than 2 h. Table 1 shows the comparison of existing human-like robotic hands.

## 2. Materials and Methods

In the human hand mechanical system, locomotion is organized by muscles, tendons, tissues, bones, and ligaments. It is the best example and benchmark for designing an anthropomorphic hand [36]. We believe that the human hand’s biomechanics is the key to hand dexterity and can be greatly replicated with a close biomimetic design. We build our robotic hand 3D model with reference to the real human hand.

### 2.1. Biological Finger Joint

Finger joints are constituted of bones, ligaments, synovial cavities, and bursae. They also control the range of motion and speed of the joints. We choose to duplicate bones and ligaments only, as synovial cavities and bursae play less important roles in finger locomotion.

Human bones are irregular in shape [37]. In our previous work, we proposed to first obtain the surface model of the target hand via 3D scanning, and then generate 3D mesh models of bones based on our fast template matching method [32,33,35].

**Figure 2:**
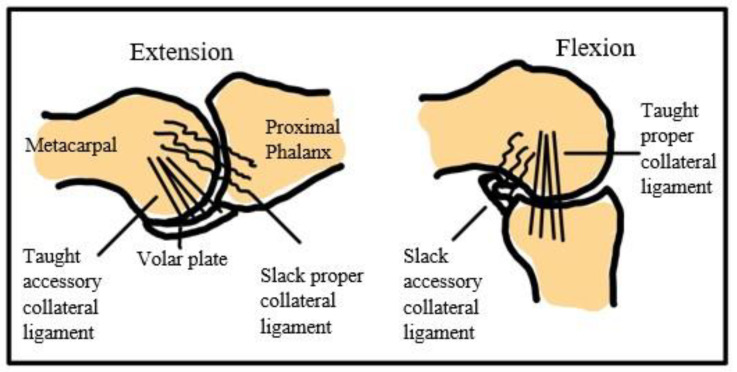
Finger joint and ligament.

A typical MCP finger joint consists of two neighbor bones and three ligaments, named the proper collateral ligament (PCL), accessory collateral ligament (ACL), and volar plate. During extension, the PCL relaxes and the ACL is taut. During flexion, the PCL is taut and the ACL relaxes. The volar plate prevents hyperextension (Figure 2). The elasticity of the ligament will help bones return to their original position.

**Figure 3:**
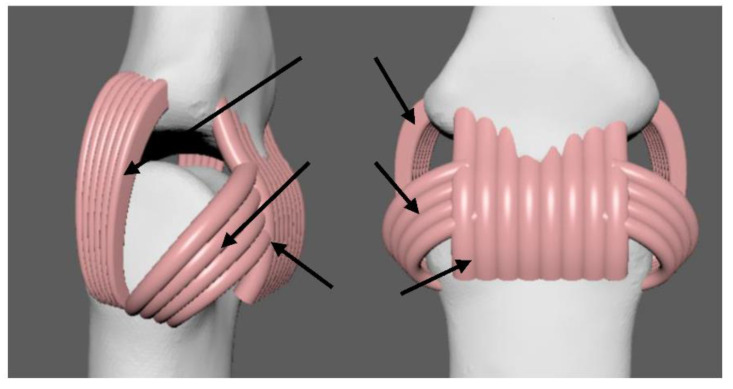
Our robotic hand joint and ligament.

The elongation of a human ligament is 68%. However, the bones are solid with no elasticity. In the traditional method of producing robotic hands, they are made by different materials and linked by a third material such as super glue or screws [28,29]. With the latest 3D modeling and 3D printing technology, we are able to generate a precise 3D-modelled hand and print different hand parts together. From Figure 3, we place multiple 0.5 mm diameter curved cylinders side by side to simulate the ACL and volar plate. We use multiple flattened 0.3 mm diameter curved cylinders side by side to simulate the PCL. An artificial ligament with too large an elongation, such as a rubber band or silicone rubber, leads to the loss of the bones’ position during moving. We choose Formlabs^TM^ Elastic 3D printing resin (RS-F2-ELCL-01) as it has a similar elongation to a human ligament. Table 2 shows the comparison of three different materials with a human ligament.

The high elongation at failure of Formlabs^TM^ Elastic 3D printing resin (RS-F2-ELCL-01) suggests that it allows the ligaments and volar plate to deform easily while flexing the finger. Otherwise, if the elongation of the material is poor, it is too difficult for the finger to bend, and the ligaments and volar plate would tend to break within a few bending movements. Another reason for us to choose Formlabs^TM^ Elastic 3D printing resin (RS-F2-ELCL-01) is because the printed product can easily change its shape when force is applied and go back to its original sharp when released. This is an important property of a tendon, be it an extensor or a flexor tendon. Some robotic hands used materials such as nylon wire and Bowden cable as their tendon; however, we would like to use a single material for the entire robotic hand, including the tendon, thus we 3D-printed the elastic tendons. However, there are some problems with elastic tendons, including the fact that its strength is reduced significantly. Nevertheless, we could still achieve the basic function of a robotic hand, showing that our idea is workable.

**Figure 4:**
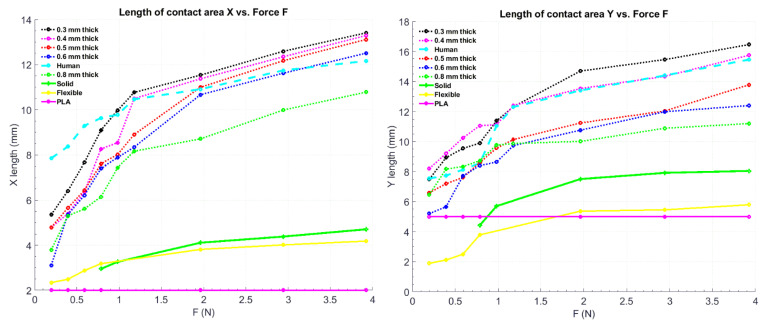
Graphical representation of the deformation of Formlab^TM^ elastic 50 A material under pressure [33].

To demonstrate that the proposed structure can effectively simulate the deformability of the human hand, we adopt the deformation curve (Figure 4) as the measure of deformability and compare the proposed structure with other methods in one of our previous works [33].

### 2.2. Biomechanical Transmission System

The hand has a complex muscle synergism. For example, the motion of each finger is handled by at least 10 muscles located at the palm and forearm [39]. These muscles actuate the bones through the tendons. Hand dexterity is a personal property. The tendon system in the human hand is different for each individual person, thus creating various ranges of motion [40].

**Figure 5:**
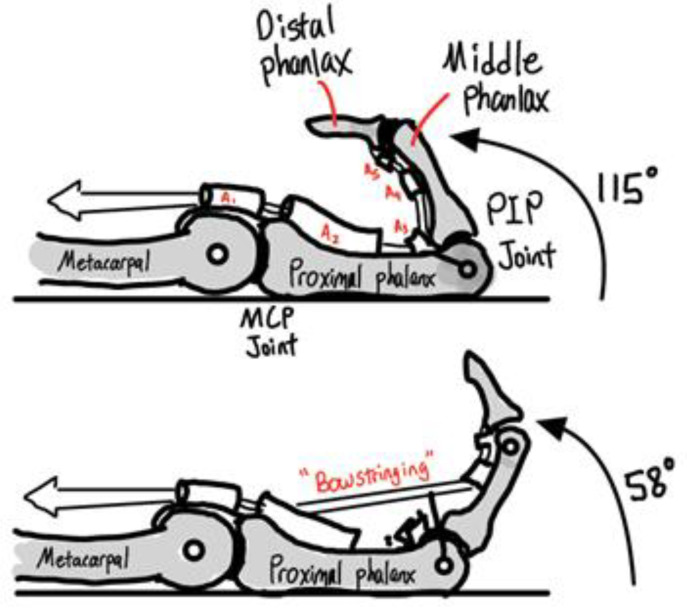
Finger pulleys’ position and usage.

The flexor pulley system of the hand is a complex structure that co-ordinates flexion of the digits. Figure 5 below shows annular pulleys A1 to A5 and their usage for finger motion [41].

**Figure 6:**
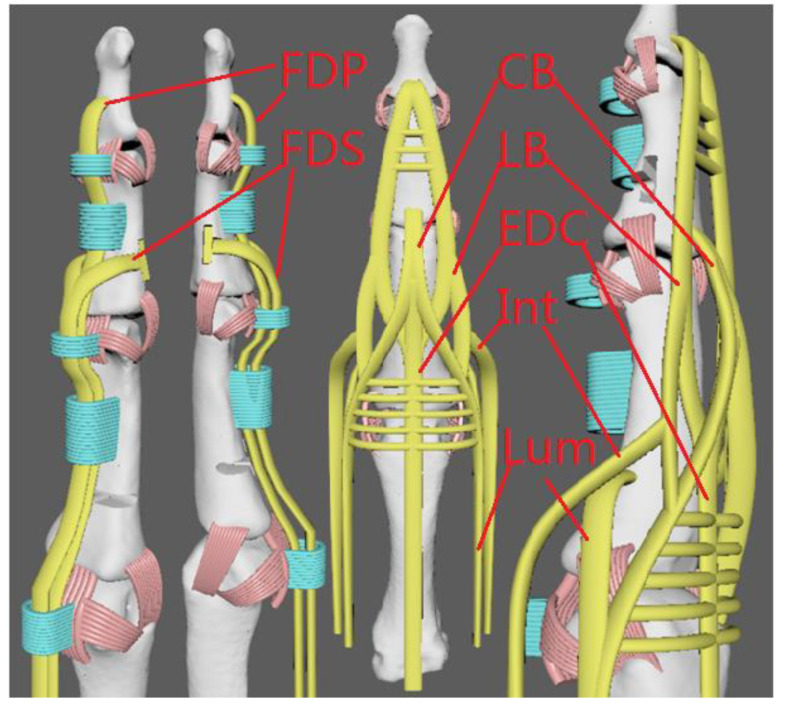
Our robotic hand tendon and pulley system.

We use multiple flattened 0.7 mm diameter curved cylinders one layer after another to simulate annular pulleys (refer to Figure 6, green part).

We design our tendon system following the real human tendon system. Flexion of the fingers is produced by two long muscles, the flexor digitorum profundus (FDP) and flexor digitorum superficialis (FDS). The FDP is connected with distal phalanges, which mainly control the flexion of DIP and PIP joints. The FDS is connected with intermediate phalanges, which mainly control the flexion of PIP and MCP joints. Finger extensor contains the extensor digitorium communis (EDC), lateral band (LB), central band (CB), and interossei (Int) and lumbrical (Lum). Lum is connected with proximal phalanges, which mainly control the addiction and abdication of joints. The joints are surrounded by soft skin and tissues. When the hand grasps, the tissue in contact deforms itself to create a greater touch area, thus leading to a firmer grip. The purposes of these complex designs are to replicate the anatomy of a human finger, whereby the tendons are similar to those of a human hand. With a realistic anatomy of a human finger, the shape and functionality of the hand could be replicated. Thus, we can apply the experience with human hands when designing and controlling the proposed robotic hand. Secondly, using a single material for printing can reduce the complexity of having various materials, as this makes it much simpler for manufacturing. Lastly, the hand is made from a soft material, where it would be compliant and would conform to the environment, making it safer for both surroundings and the users.

The features offered using these complex designs similar to the robotic hand include the following:

1. Simple input as actuation (requires prime mover to pull the tendon to move the fingers);
2. Lightweight (lack of gears and mechanisms allows a lighter design);
3. Soft material (compliant and safer);
4. Single material (easy to manufacture);
5. Bio mimetic (more natural to view and use).

**Figure 7:**
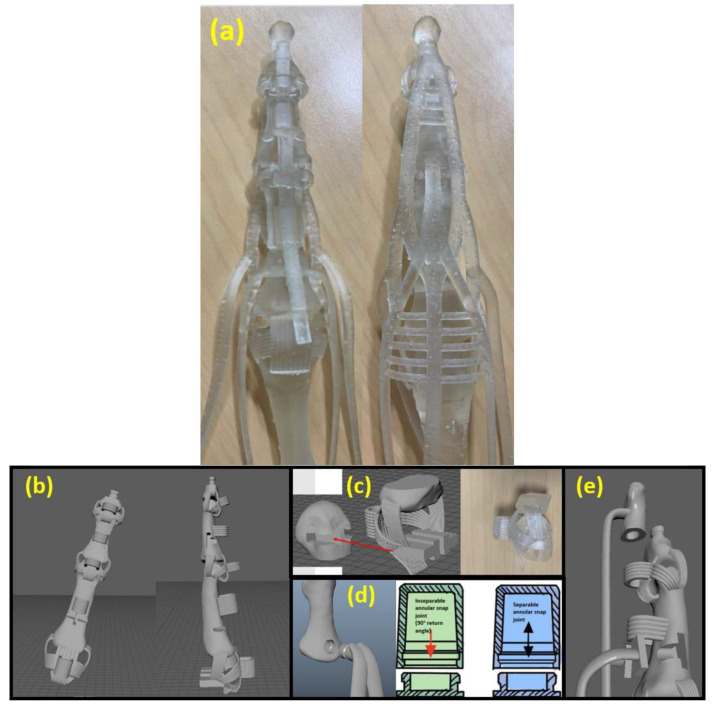
(**a**) Front and back view of our 3D-printed robotic finger; (**b**) 3D model of the index finger; (**c**) lap joint example for ligament connection at the MCP; (**d**) annular snap-fit joint for a extensor tendon; and (**e**) annular snap-fit joint for a flexor tendon.

The final 3D-printed result is shown in Figure 7a.

The final 3D-printed result is shown in Figure 7a. We 3D-printed the ligament connection at the distal interphalangeal (DIP) and proximal interphalangeal (PIP) joint, thus it is printed as one piece; its 3D model is shown in Figure 7b. For ligament connection at the metacarpophalangeal (MCP), we used lap joint design. Figure 7c shows how we connected the ligaments and bone together. We could see in Figure 7c that the proper collateral ligament (PCL) and accessory collateral ligament (ACL) are connected to a T-sharp plug, which could be inserted into the T-sharp port located at the top of the metacarpal bone. We could use this type of joint because the force direction applied along the tendon would always be perpendicular to the plug motion direct, so there is no need to worry about whether the joint would slide open while the robotic hand operates, as the friction between the elastic material is sufficient to hold the joint. To connect extensor tendons to the bone, we used an annular snap-fit joint. As shown in Figure 7d, the tip of the extensor tendon is the male part of the snap-fit joint, and the bone to which it is connected is the female part of the snap-fit joint; when the snap is actuated, it holds the bone and tendon firmly together. To connect the FDP and FDS, we made use of both the snap-fit joint and lap joint. As shown in Figure 7e, we actually separated the distal phalange into two parts; we modelled the FDP as a whole with the upper distal phalange bone and printed it as one piece, and then connected the upper and lower distal phalange with a snap-fit joint. For the FDS, we used a lap joint by making the tip of the FDS a rectangular sharp plug, which was then placed into the T-sharp port located at the middle or proximal phalange. The benefit of the various kinds of joint is that no other adhesive is needed for the whole assembly process, thus reducing the time, skill, and effort required to assemble the robotic hand.

### 2.3. Dynamic Mode and Actuation System

**Figure 8:**
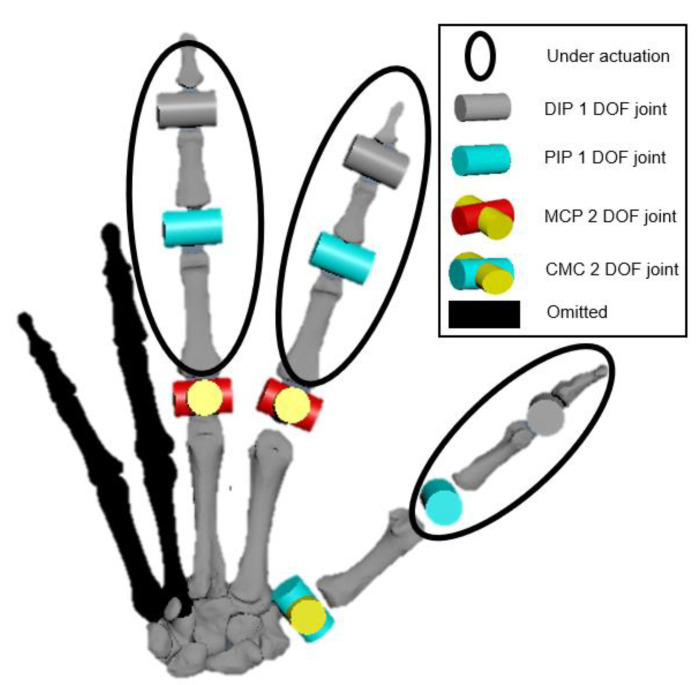
The simplified 9-DOF model of the proposed hand.

In this first prototype, we chose to make a three-finger robotic hand to verify our design, as a fully replicated human hand would lead to a bulky and complex actuation system [27,30,42]. The under-actuation method will reduce the number of actuators, but lead to the loss of independent control for each joint [3,28]. Previous research showed that the distal interphalangeal (DIP) and proximal interphalangeal (PIP) joints work like an under-actuated system [43]. In this proposed design, for each finger, one tendon (FDS) is used to control the DIP and PIP joints at the same time. The EDC tendon is used to extend the finger for recovery. We further study the maximum range of motion (ROM) required to achieve 33 gestures from grasping taxonomy and reduce the two least important DOFs from the ring finger’s metacarpophalangeal (MCP) joints. As a result, the dynamic model of this robotic hand also has 15 movable joints, but only 8 independent DOFs plus 5 under-actuated DOFs (refer to Figure 8).

Each joint is driven by corresponding tendons. Based on Landsmeer’s model III [44], the basic equation for modeling the tendon displacement (*L*) can be simplified to a second-order polynomial approximation [45]:

(1) $$L=h\theta^{2}+b\theta$$

where *b* and *h* are constants determined by the mechanical system and *θ* is the corresponding angle rotation.

## 3. Experiments

### 3.1. Trajectories of the Fingers

Because the size of the hand and the grasped object will affect the results, the human hand used as a reference is 180 mm in length. Further, the size of grasped objects was taken from [46]. We compare the human hand ROM to our robotic hand ROM in Table 3. This robotic hand duplicates about 90% of the human maximum range of motion (refer to Table 3).

**Figure 9:**
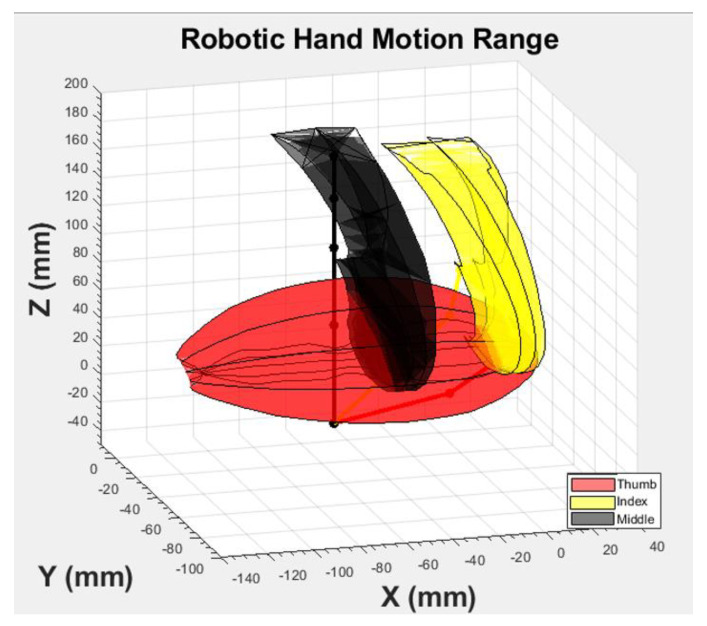
Graphical representation of the motion of the fingertips for each design during actuation.

We also recorded the trajectories of each finger and plotted them in a 3D space (Figure 9).

### 3.2. Grasping Test

**Figure 10:**
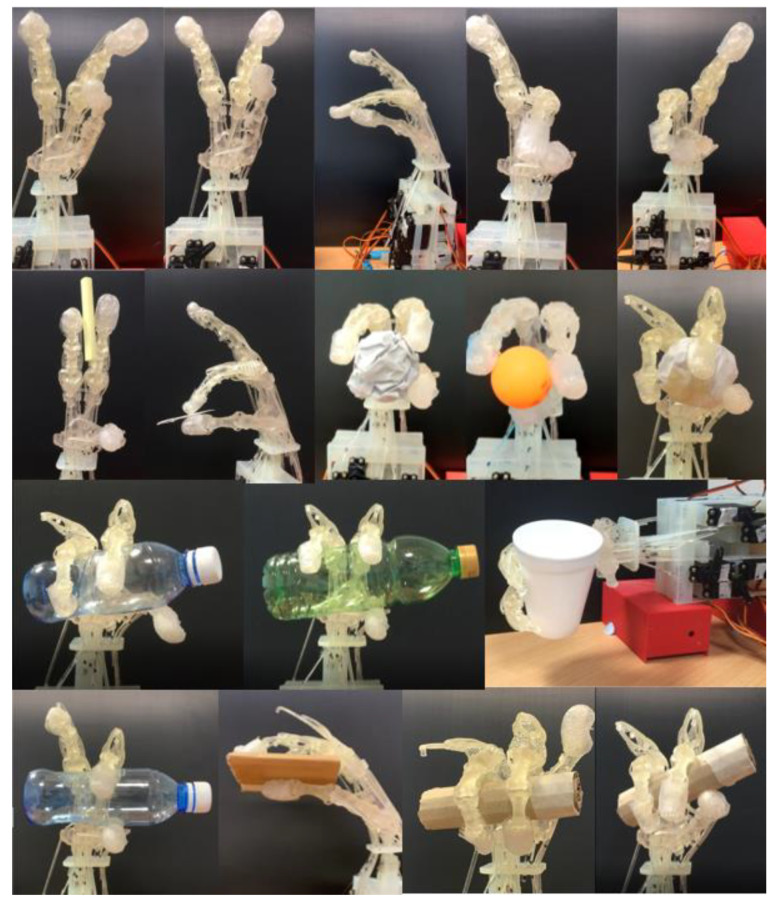
Tasks. Row 1 presents the performance of five Kapandji tests. Rows 2–4 show the robotic hand performing 12 gestures from the grasping taxonomy.

In order to evaluate the performance of our robotic hand, we performed the Kapandji test and grasping test for our robotic hand. All kinds of test cases were tested 10 times separately and all success rates were larger than or equal to or 90%. Please refer to Figure 10 for details (the test video is attached as Video S1).

## 4. Conclusions and Future Works

We have described a new method to create a single-material highly biometric robotic hand and to actuate it to achieve grip poses as well. Our robotic hand is designed based on the anatomy of the human hand. The experiments show that our method is feasible and promising. In the future, we plan to enhance the stability and robustness of our robotic hand by adding on the remaining ring and little finger, which allows the robotic hand to perform more grasping gestures. Moreover, we plan to envelope the fingers and palm area with soft tissues. An extension to the current stationary robotic hand can be added, such as a forearm and shoulder, which provides some room for movement for the robotic hand to perform more gestures that require the movement of the arm. We will search new materials that can be used as a replacement for Elastic 50 A, so as to have a stronger and stiffer robotic hand to grasp heavier objects. Lastly, we will further improve the design and structure to make it more human-like.

## Figures and Tables

**Table 1:** Comparison of human-like robotic hands.

Type	Representative Product	Appearance	Actuated Method	Joint	Tendon	Pulley System
Traditional	Shadow hand [3]	Mechanical rigid	Electric motor	Metal pin-joint	Thread	Rigid structure, robust
Highly biometric	Xu Zhe’s robotic hand [28]	Bones based	Electric motor	Crocheted ligament	Nylon thread	Laser-cut rubber sheet
Soft material	RBO hand [13]	Streamlined cylinder	Pneumatic/Fluidic motor	No	Curl	Soft body, big actuation system
Soft material + Soft actuator	Yu She’s robotic hand [34]	Streamlined cylinder	Soft actuator (Shape memory alloy)	No	Curl	Soft body, Small grip force
flexible surface + rigid palm bones	Nadine hand V4 [35]	Hand shape	Electric motor	Flexible pin-joint	Turn	Soft body, weak in joint structure
Highly biometric	Nadine hand V5 [32]	Hand shape (capable with silicone skin)	Electric motor	Bone-joint	Turn	Soft body, rigid bones
Single material	The proposed design	Hand shape (capable with silicone skin)	Electric motor	Soft bone-Joint	Turn	Soft body, soft bones

**Table 2:** Comparison of 3D-printed materials and a rubber band.

	Formlabs Flexible FLGR02	Formlabs Elastic RS-F2-ELCL-01	Rubber Band	Human Ligament [38]
Elongation	120%	160%	750–850%	68%
Tensile Strength	7.7–8.5 MPa	3.2 MPa	20–30 MPa	50–150 MPa
Hardness	80–85 A	50 A	20–30 A	N.A.

**Table 3:** Comparison of the range of motion.

Finger	Joint	Human Hand (°) [47,48]	The Proposed Design (°)
Thumb	IP	0–90	0–85
	MCP	0–70	0–83
	CMC	0–53	0–60
	Add./Abd.	−40–50	0–50
Index	DIP	0–80	0–70
	PIP	0–120	0–77
	MCP	0–90	16–110
	Add./Abd.	−20–25	−25–26
Middle	DIP	0–80	0–78
	PIP	0–120	0–92
	MCP	0–90	−19–100
	Add./Abd.	−20–25	−10–26

## Supplementary Materials

The following are available online at https://www.mdpi.com/article/10.3390/mi12091124/s1, Video S1: One Material Hand.
